# Use of an antagonist of HMGB1 in mice affected by malignant mesothelioma: a preliminary ultrasound and optical imaging study

**DOI:** 10.1186/s41747-021-00260-y

**Published:** 2022-02-08

**Authors:** Massimo Venturini, Rosanna Mezzapelle, Salvatore La Marca, Laura Perani, Antonello Spinelli, Luca Crippa, Anna Colarieti, Anna Palmisano, Paolo Marra, Andrea Coppola, Federico Fontana, Giulio Carcano, Carlo Tacchetti, Marco Bianchi, Antonio Esposito, Massimo P. Crippa

**Affiliations:** 1 Department of Diagnostic and Interventional Radiology, Circolo Hospital, ASST-Sette Laghi, Insubria University, Via Guicciardini 9, 21100, Varese, Italy; 2 Department of Medicine and Surgery, Insubria University, Varese, Italy; 3 Chromatin Dynamics Unit, Division on Genetics and Cell Biology, San Raffaele Scientific Institute, Milan, Italy; 4 Department of Radiology, San Raffaele Scientific Institute, Milan, Italy; 5 Experimental Imaging Center, San Raffaele Scientific Institute, Milan, Italy; 6 ISTOVET, Besana in Brianza, Monza e Brianza, Italy; 7 Vita-Salute San Raffaele University, Milan, Italy; 8 Department of Diagnostic Radiology, Giovanni XXIII Hospital, Milano-Bicocca University, Bergamo, Italy; 9 Department of General, Emergency and Transplants Surgery, Circolo Hospital, ASST-Sette Laghi, Varese, Italy

**Keywords:** HMGB1 protein, Mice (inbred BALB C), Mesothelioma (malignant), Optical imaging, Ultrasonography

## Abstract

**Background:**

Malignant mesothelioma (MM) is an aggressive tumor, with a poor prognosis, usually unresectable due to late diagnosis, mainly treated with chemotherapy. BoxA, a truncated form of “high mobility group box 1” (HMGB1), acting as an HMGB1 antagonist, might exert a defensive action against MM. We investigated the potential of BoxA for MM treatment using experimental 40-MHz ultrasound and optical imaging (OI) in a murine model.

**Methods:**

Murine MM cells infected with a lentiviral vector expressing the luciferase gene were injected into the peritoneum of 14 BALB/c mice (7 × 10^4^ AB1-B/c-LUC cells). These mice were randomized to treatment with BoxA (*n* = 7) or phosphate-buffered saline (controls, *n* = 7). The experiment was repeated with 40 mice divided into two groups (*n* = 20 + 20) and treated as above to confirm the result and achieve greater statistical power. Tumor presence was investigated by experimental ultrasound and OI; suspected peritoneal masses underwent histopathology and immunohistochemistry examination.

**Results:**

In the first experiment, none of the 7 controls survived beyond day 27, whereas 4/7 BoxA-treated mice (57.1%) survived up to day 70. In the second experiment, 6/20 controls (30.0%) and 16/20 BoxA-treated mice (80.0%) were still alive at day 34 (*p* = 0.004). In both experiments, histology confirmed the malignant nature of masses detected using experimental ultrasound and OI.

**Conclusion:**

In our preclinical experience on a murine model, BoxA seems to exert a protective role toward MM. Both experimental ultrasound and OI proved to be reliable techniques for detecting MM peritoneal masses.

## Key points

- BoxA wild type, an antagonist of high mobility group box 1, seems to exert a protective role on malignant mesothelioma in a murine animal model.
- Experimental 40-MHz ultrasound can characterize, quantify, and monitor peritoneal malignant mesothelioma masses.
- Optical imaging can detect abdominal masses according to their luminescence intensity in an animal model study.

## Background

Malignant mesothelioma (MM) is a rare tumor arising from the mesothelial cells lining the pleural and peritoneal cavities, or less commonly from the pericardium and the tunica vaginalis of the testis [1]. The association of pleural MM with asbestos exposure is well established [2]. In the USA and Europe, up to 80% of MMs occur in the pleura since asbestos, after inhalation in the lungs, reaches the pleura via the lymphatic system. MM prognosis remains very poor, with a median survival of 6–12 months and a 5-year survival lower than 5% [3]. Chest computed tomography represents the imaging technique of choice to evaluate MM, including the extent of primary tumor, local invasion, intrathoracic lymphadenopathy, and extrathoracic spread [4].

Surgery in combination with radiotherapy and chemotherapy can be used for otherwise healthy patients with early disease stage, but most patients have unresectable disease at the time of diagnosis and are often treated only with palliative chemotherapy [5]. Pleural MM is resistant to chemotherapy, although the combination of pemetrexed and cisplatin, the most commonly used regimen, leads to an overall survival benefit of about 11 weeks [6]. Intra-arterial chemotherapy, previously employed in hepatic tumors using catheter-port systems [7], has been also used in MM [8]. Nowadays, new forms of treatments are under investigation to improve the prognosis, such as immunotherapy [9]. In previous preclinical studies on MM, it was well established that the nuclear protein “high mobility group box 1” (HMGB1) plays a crucial role in the inflammatory pathway and in MM development [10, 11]. BoxA is a truncated form of HMGB1, which acts as an HMGB1 antagonist [12, 13].

Ultrasound and optical imaging are used in experimental studies to validate preclinical models of various malignant neoplasms, providing non-invasively structural and functional information and allowing also a reduction of experiments and sacrificed animals [13–18].

The aim of our study was to investigate the protective role of BoxA for MM treatment in an experimental setting in a murine model, using experimental ultrasound and optical imaging (OI) as techniques for detecting and monitoring peritoneal implants of MM cells, using histology as a reference standard.

## Methods

The design of this experimental study was based on the survival of mice with induced MM comparing controls and BoxA-treated mice and the accuracy of ultrasound and OI in detecting peritoneal masses.

Murine MM AB1cells were obtained from Cell Bank Australia and cultured in RPMI 1640 (Life Technologies, New York, USA) supplemented with 5% volume/volume fetal bovine serum (Life Technologies), 2 mM l-glutamine and 100 U/mL penicillin/streptomycin and subsequently intraperitoneally injected in “Bag ALBino” (BALB/c) mice. Masses grown in BALB/c mice were explanted and the procedure to obtain AB1 cells expressing the gene for the luciferase enzyme (AB1-B/c-LUC) cells was previously described [13].

HMGB1 contains two deoxyribonucleic acid-binding domains, named BoxA (BoxA alone behaves as a HMGB1 competitor [19] while it contains an epitope that promotes inflammation) and an acidic tail. BoxA corresponds to amino acids 2–89 of HMGB1, where amino acid 1 is methionine and is removed both in mammalian HMGB1 and in BoxA; the sequence of this segment of HMGB1 is identical in all mammals [11].

Animal experiments have been reviewed and approved by the Institutional Animal Care and Use Committees of Ospedale S. Raffaele and Istituto di Ricerche Farmacologiche “Mario Negri,” which include ad hoc members for ethical issues. Animals were housed in the Institutes Animal Care Facilities, which meet international standards. Certified veterinarians who are responsible for health monitoring, animal welfare supervision, experimental protocols, and procedure revision regularly checked them, in both institutions.

The following two experiments were performed.

*First preliminary experiment*. Fourteen mice were intraperitoneally injected with 7 × 10^4^ AB1-B/c-LUC cells (in 500 μL), and on the same day, the 1st OI was performed (intraperitoneal 200 μL luciferin) to confirm the success of injection. The mice were randomized into two groups (*n* = 7 + 7) and treated intraperitoneally, every other day for 28 days, as follows: 7 mice with BoxA wild type (400 μL, 2 mg/mL = 800 μg, corresponding to 32 mg/kg); 7 mice with 400 μL of phosphate-buffered saline (controls).

*Second experiment.* Forty mice were injected with MM cells and divided into two groups (*n* = 20 + 20), which were treated intraperitoneally with BoxA or phosphate-buffered saline as above every other days for 33 days. Some mice that survived to the second experiment were rechallenged after 2 months with murine MM cells to confirm the tumor rejection. The second experiment was developed to increase the robustness of the preliminary data, increasing the number of the mice.

Tumor response and survival were assessed in both experiments using experimental ultrasound- and OI-based parameters.

### Ultrasound protocol

Ultrasound examination was performed using a 40-MHz linear probe (Vevo 2100, Fujifilm Visualsonics, Toronto, Canada), both using freehand and fixed support. The ultrasound protocol was based on the study of the abdomen of the mouse divided into 9 quadrants which were likely to correspond to those of human and each quadrant was given a number (from 1 to 9): right hypochondrium, epigastric region, left hypochondrium, right flank, mesogastric region, left flank, right iliac fossa, hypogastric region, and left iliac fossa.

Tumor response was evaluated with ultrasound through the presence or absence of masses; number, volume, distribution, growth and shrinkage of mass, and presence or absence of ascites were also considered. All the following mass parameters were evaluated using brightness-mode ultrasound and color Doppler: echogenicity, shape, margins, vascularization, and diameter. In the first experiment, ultrasound was performed on day 5, 22, and 32 and in the second experiment, instead, on day 30.

### Optical imaging protocol

The system was equipped with a low noise, back-thinned, back-illuminated charge-coupled-device camera cooled to − 90 °C (quantum efficiency in the visible range above 85%). Before the OI procedure, each mouse received an intraperitoneal injection of 6 g of luciferin (d-luciferin potassium salt, Perkin Elmer, Milan, Italy) per kilogram of body weight. Luciferase oxidizes luciferin to generate light, which is produced because the reaction forms oxyluciferin in an electronically excited state. The reaction releases a photon of light as oxyluciferin goes back to the ground state [20] and can be quantified by OI.

During image acquisition, the animals were kept at 37 °C and under gaseous anesthesia (2–3% isoflurane and 1 L/min O_2_). After luciferin injection, OI was performed from 0 to 30 min by acquiring images every 2 min in order to detect the highest OI signal, with the following technical setting: exposure time “auto”; binning8, focal ratio 1; and a field of view of 13 cm (field C).

Optical imaging was performed using the Living Image 4.4 software (PerkinElmer’s IVIS Spectrum Pre-clinical in Vivo Imaging System, Milan, Italy) by measuring the total light flux (photons/s) in a region of interest which included the abdomen and excluded limbs and testicles of mice. OI was also performed to evaluate the presence or absence of masses according to their luminescence intensity. The timing of OI execution was day 1, 6, 14, 20, 27, 31, 41, and 48 for the first experiment and day 1, 6, 13, 30, 34, 61, 68, 76, and 84 for the second experiment.

### Comparison between ultrasound and OI

Detection (presence/absence) of masses was recorded for both techniques and the findings were compared using pathology as a reference standard. Suspicious masses and samples from abdominal organs were histopathologically and immunohistochemically studied after the mice were sacrificed [13]. For histopathological analysis, samples of explanted tumor masses were fixed in 10% neutral buffered formalin for 24–48 h, processed with a Tissue Processor (Leica, Buccinasco-Milan, Italy) and paraffin embedded. Sections of 4 μm were cut, stained with hematoxylin-eosin, and evaluated under a light microscope.

### Statistical analysis

Contingency tables and Fisher exact test were used to assess difference both in the first and in the second experiment between the two mouse populations both for mortality and for the presence of masses at ultrasound evaluation. Concordance between mass detection at imaging (ultrasound and OI) and histology was investigated using simple contingency tables. A two-tailed *p* value lower than 0.05 was considered significant. Statistical analyses were performed using the SPSS software, version 25.0 (IBM, Armonk, New York, USA).

## Results

### Mouse survival

**Fig. 1:**
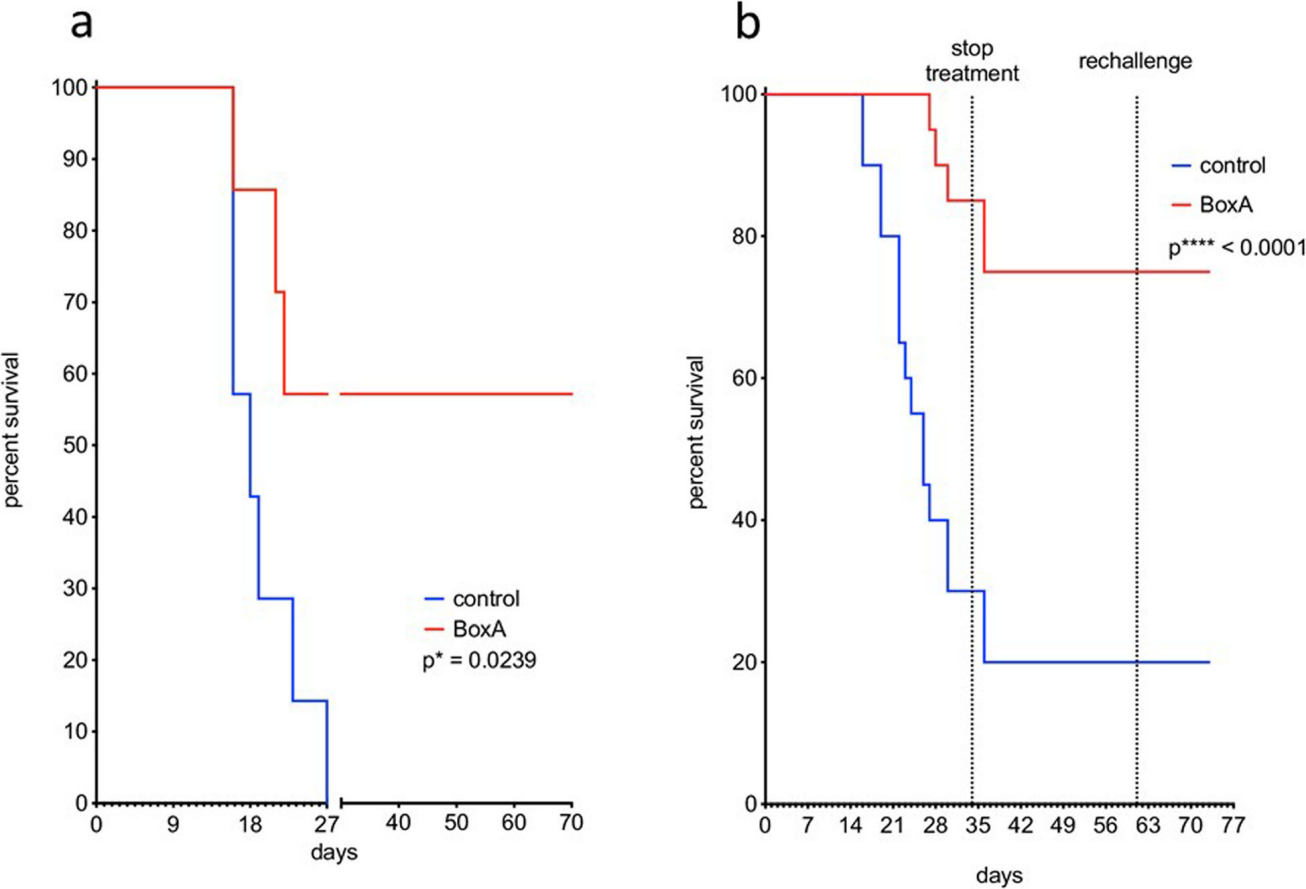
Mouse survival in the first and second experiments. **a** The two mouse groups (*n* = 7 + 7) of the first experiment were BoxA-treated (red line) and phosphate-buffered saline-treated, *i.e.*, controls (blue line). Their survival was evaluated up to the 70th day. BoxA-treated mice showed the greatest survival rate: 57.1% at the 70th day after the inoculation of malignant mesothelioma cells. **b** In the second experiment, the survival curves of controls and BoxA-treated mice were evaluated up to the 34th day after the inoculation and up to day 73 both for rechallenged and non-rechallenged mice: BoxA-treated mice had a highly significant survival rate than controls (same line colors as for panel **a**)

In the first preliminary experiment, none of the 7 control mice survived beyond day 27 whereas 4 out of 7 BoxA-treated mice survived up to day 70 (57.1%) (Fig. 1a). They survived after treatment suspension and no malignant masses were found at necropsy. In the second experiment, 6 out of 20 control mice (30.0%) and 16 out of 20 BoxA-treated mice (80.0%) were still alive (Fig. 1b) at day 34, when the treatment was stopped (*p* = 0.004, Fisher exact test). Two days later, 2 control and 2 BoxA-treated mice died, bringing the survival rate to 20% for control mice and 75% for BoxA-treated mice. Two control and 6 BoxA-treated mice were rechallenged at day 61 with murine MM cells. Both the rechallenged and the non-rechallenged mice of both groups survived to the end of the experiment, suggesting tumor rejection.

### Ultrasound evaluation

**Fig. 2:**
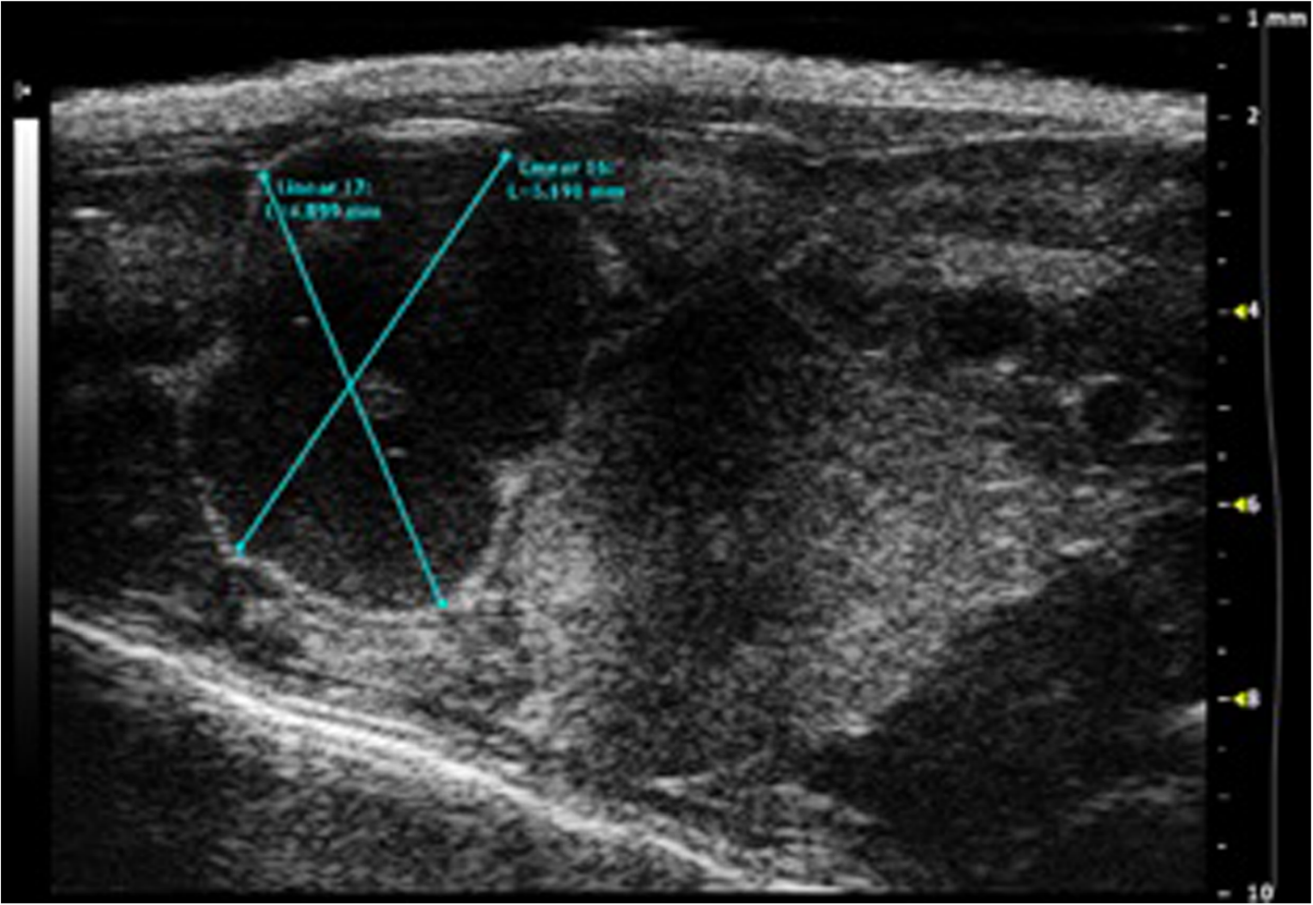
Ultrasound image showing a large mass in a phosphate-buffered saline-treated mouse (control) in the abdomen (right iliac fossa)

In the first experiment, ultrasound showed the presence of masses in mice’ abdomen as follows: there were 4 masses in the 7 BoxA-treated mice (4/7 mice with masses) and 7 masses in the 7 controls (7/7 mice with masses) (*p* = 0.192, Fisher exact test). In the second experiment, ultrasound showed the presence of 1–9 abdominal masses in 5 of 20 BoxA wild type-treated mice and in 16 of 20 controls (*p* = 0.001, Fisher exact test) (Fig. 2). At the end of the study, all the detected masses appeared as hypoechoic lesions, oval shaped, with well-defined margins, highly vascularized at color Doppler with a diameter variable from 0.5 to 7.5 mm.

### Optical imaging evaluation

**Fig. 3:**
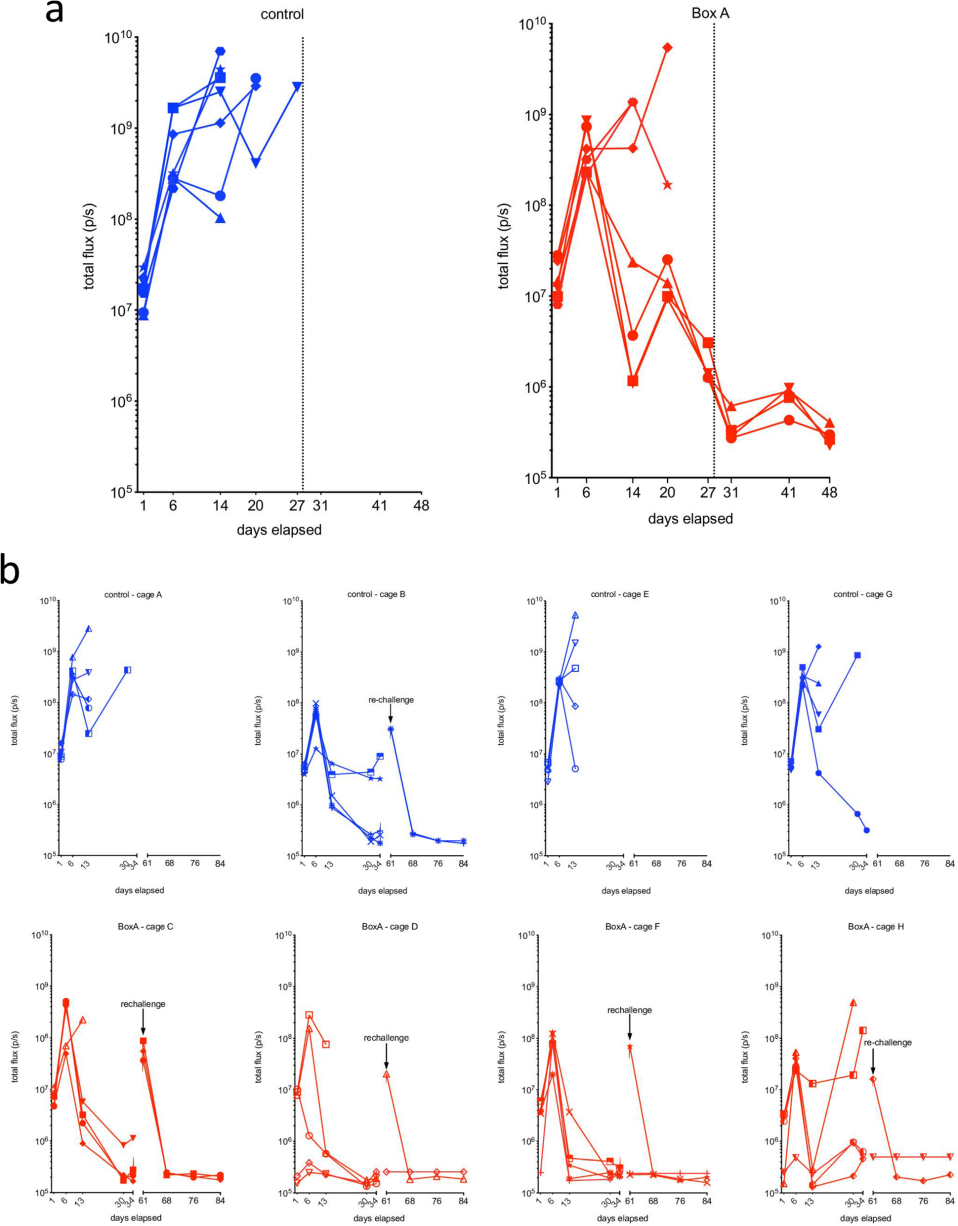
OI curves in the first and second experiments. **a** In the first experiment, phosphate-buffered saline-treated mice (blue line) were studied at five timepoints with OI, after which all mice had been sacrificed. BoxA-treated mice (red line) were studied for 8 timepoints, since 4 out of 7 had survived. In the control mice from the first to the last OI acquisition, there was an increase of the signal for the growth of tumor cells’ masses due to a significant tumor progression. In BoxA-treated mice that did not survive, tumor progression was as observed in control mice, whereas in the surviving mice we did not observe any tumor onset. **b** In the second experiment, both control (blue line) and BoxA-treated mice (red line) were initially studied for 5 timepoints. The results were similar to those showed in panel **a**, in that most control mice and a fraction of BoxA-treated mice developed tumors, whereas the surviving mice did not. At day 61, two controls and 6 BoxA-treated mice were rechallenged with murine MM cells and studied by OI for another 12 days in which remained tumor-free

In the first experiment, at the 1st scan, OI evaluation showed positive luminescence suggestive of the presence of masses (1-9) of MM cells in the abdomen of the mice. We therefore started the treatment with either phosphate-buffered saline (control) or BoxA (Fig. 3a). At the 2nd scan, all mice had an increase of the signal of at least one order of magnitude (Fig. 3a). From the 2nd scan to the 5th scan, all controls and 3 out of 7 BoxA-treated mice had either an increase in the signal or a marginal reduction of less of an order of magnitude. None of the controls survived for the 6th scan. However, 4 out of 7 BoxA-treated mice had a reduction of the signal of at least three orders of magnitude and survived for the 6th scan.

**Fig. 4:**
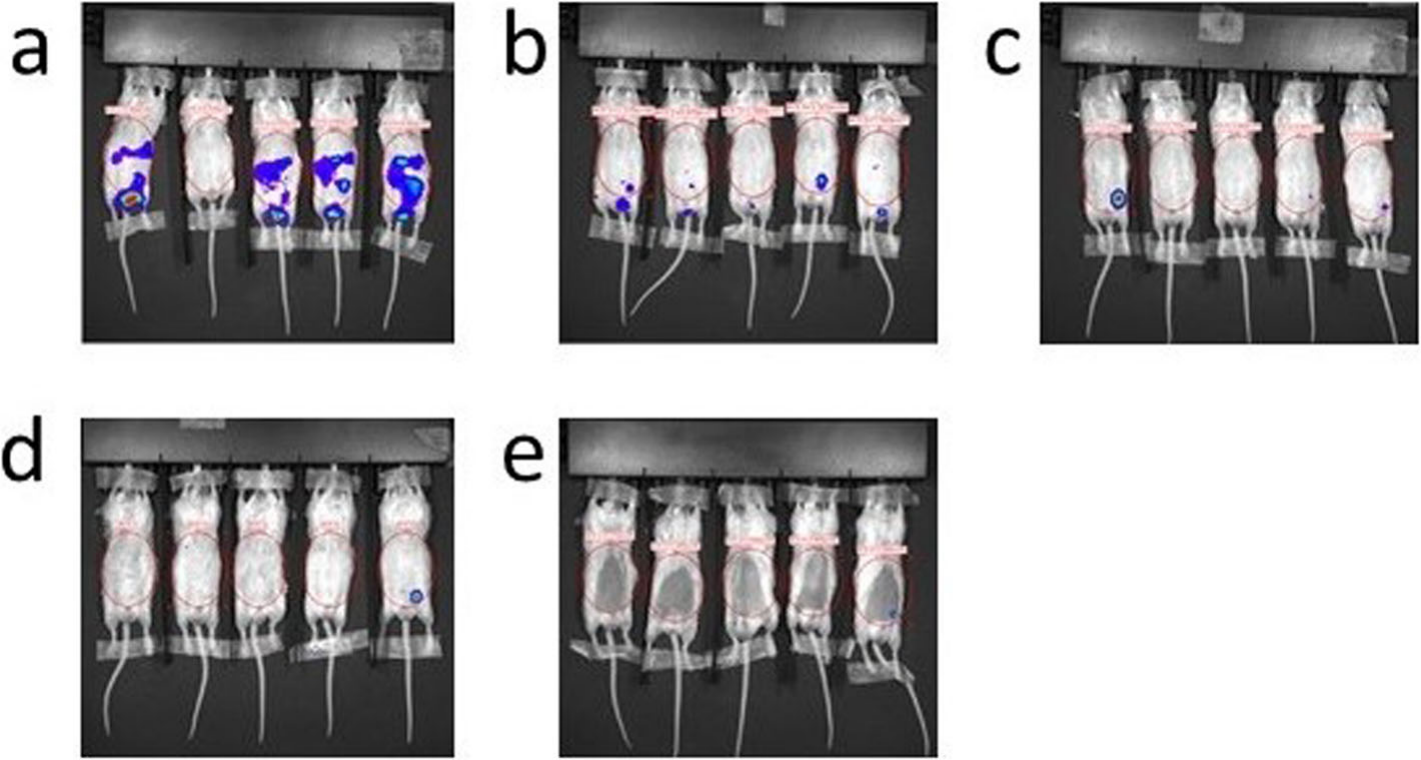
Optical imaging during acquisition. Five representative BoxA-treated mice of the second experiment. Panels **a**, **b**, **c**, **d**, and **e**: day 1, 3, 6, 30, and 34 from injection, respectively. From **a** to **e**, a reduction of the OI signal due to the protective role of BoxA treatment can be observed

In the second experiment, at the 1st scan, OI evaluation was positive for all the 20 BoxA-treated mice and all 20 controls (Fig. 3b). All the control values were between 1.59 × 10^7^ photons/s (photon flux) and 2.83 × 10^6^ photons/s, while the BoxA wild type-treated group values were subdivided into two groups: 15 of the 20 (75%) BoxA-treated mice had values between 1.08 × 10^7^ and 2.88 × 10^6^ photons/s, while the other 5 mice had values one order of magnitude lower than the other (< 2.52 × 10^5^ photons/s). At the 2nd scan, 20 of 20 controls and 16 of 20 (80%) BoxA-treated mice showed a growth of the signal from the first scan of at least one order of magnitude. From the 2nd to the 5th scan, 4 of 20 (20%) controls showed an increase of the signal of at least one order of magnitude, while in 6 of the 20 (30%) controls there was a stable reduction of the signal of at least one order of magnitude; these 6 were the only controls which survived for the 6th scan. Moreover, 2 of 20 (10%) BoxA-treated mice showed an increase of the signal of at least one order of magnitude, while in 16 of the 20 (80%) BoxA-treated mice, there was a stable reduction of the signal of at least one order of magnitude; all of them survived to the 6th scan (Fig. 4).

### Comparison between ultrasound and OI

**Fig. 5:**
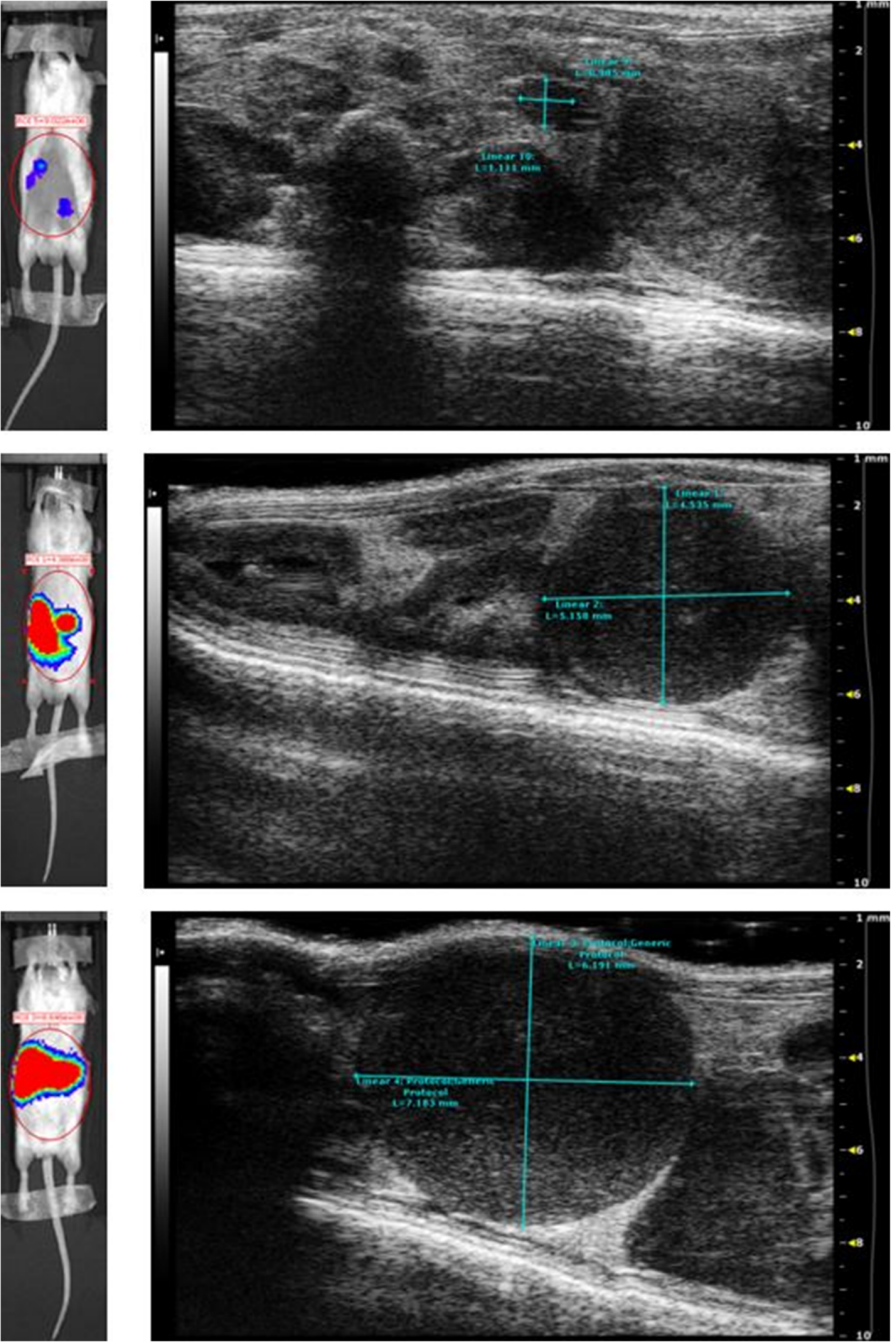
In the cases here shown, both ultrasound and optical imaging showed mass presence at a single timepoint in three different mice

Among the BoxA-treated animals, 3 mice were positive to OI only in the site of inoculation of MM cells, treatment and luciferase, and were negative to ultrasound; a single mouse was negative to both OI and ultrasound; and a single mouse was positive both to OI and ultrasound. Among the controls in all the 3 mice which were positive to “spectrum in vivo imaging system,” there were masses at ultrasound scan (Fig. 5).

### Histopathology

Explanted tumor masses showed a sarcomatoid-epithelioid aspect similar to human MM with significant vascularization and poor necrosis. In both experiments, mice which were both negative to OI and ultrasound imaging were also negative to the histological analysis, while mice which were positive to OI and ultrasound were also positive to histology.

## Discussion

Malignant mesothelioma is a relatively rare tumor, but incidence and mortality trends are leveling off [21]. The overall prognosis for patients with MM is poor, with a median survival of 6–12 months after diagnosis [22], mainly due to the late diagnosis, which generally does not allow a radical therapy. Therefore, new treatments need to be found to improve the prognosis. Chest computed tomography is the imaging modality of choice to evaluate MM and demonstrates the extent of primary tumor, local invasion, intrathoracic lymphadenopathy, and extrathoracic spread.

Immunotherapy represent an innovative and promising strategy for the treatment of MM, and various experimental drugs have been proposed such as monoclonal antibodies (*e.g.*, against CTLA4, PD-1, and PD-L1) [23] and mesothelin-targeting agents (anticancer antibodies/antibodies-drug conjugates; mesothelin-targeting vaccines; mesothelin-targeting recombinant T cells). As explained in the “Background” section, in the field of groundbreaking treatments, BoxA, an HMGB1 antagonist [24, 25], could be a new strategy for immunotherapy.

According to the findings of this study, BoxA showed encouraging perspectives.

In the first preliminary experiment, BoxA-treated mice survived after treatment suspension and no malignant masses were found at necropsy. In the second experiment, BoxA-treated mice also survived to the end of the experiment, suggesting a protective role of BoxA.

The beneficial action of BoxA wild type has been demonstrated and its mechanism has recently been clarified [26]. BoxA triggers a mechanism named “immunogenic surrender,” an immunosurveillance process in which innate immunity plays a paramount role through macrophages and activates acquired immunity, leading to tumor rejection.

Monitoring tumor growth in individual animals with the techniques described in this study has led to three critical observations linking BoxA to acquired immunity:

- The time of regression of the malignant masses coincides with the time of the adaptive response.
- The BoxA protective effect is all or nothing, like the stochastic efficacy of adaptive immunity.
- The protective role of BoxA wild type remains after the suspension of treatment, in the form of immune memory cells.

Indeed, the adaptive immune system has been shown to be activated to produce an acquired immunity through an unsuspected immunosurveillance axis, involving CXCR4 and CD47 molecules [25]. This immunotherapy approach could be applied to other HMGB1 overexpressing tumors such as, for instance, colorectal, as it has been shown [24].

Murine models of cancer provide a critical link between fundamental discoveries about the efficacy of new diagnostic imaging approaches or new therapeutic treatments, allowing clinical translation [27–30]. Murine models require various experimental imaging procedures which have different advantages and disadvantages relating to the biological profile.

Anatomical information can be acquired with magnetic resonance imaging (MRI), computed tomography, and ultrasound. MRI has higher accuracy but longer acquisition times than ultrasound, which is a real-time and less expensive imaging technology. Functional and metabolic information can be acquired with positron emission tomography, hyperpolarized MRI, and OI. Computed tomography provides excellent anatomical information at high resolution despite lack of sufficient soft tissue contrast. However, computed tomography acquires data very fast compared to positron emission tomography or MRI and it can easily be installed within existing laboratories.

Ultrasound was also chosen to characterize and quantify the malignant masses; it permits non-invasive and real-time visualization of organs and tissues. This technology is adapted for use in mice through the utilization of higher frequency transducers (20–50 MHz), allowing high-resolution imaging and an adequate penetration for anatomical and functional real-time information about animal models [30]. Ultrasound imaging captures dynamic, real-time images with optimal spatial resolution obtaining quantitative structural and functional information. Commercial ultrasound systems are less expensive than other imaging technologies. Other advantages in preclinical studies are the short time of execution, which allows the examination of a large number of mice, and the reproducibility of quantitative measurements (lengths, volumes, flow parameters, and enhancement parameters).

Among all the experimental imaging technologies, OI was chosen to study the dynamic changes in biology over time, but it is not a completely quantitative imaging method, as the emitted light is prone to attenuation due to overlying tissue [31]. OI is used to assess the viability of tumor cells in response to BoxA treatment by bioluminescence. In normal conditions, mammalian cells do not emit light, while transformed viable tumor cells emit light when injected with luciferine; thus, the signal-to-noise ratio of OI is so accurate that OI is an extremely sensitive preclinical approach to assess pathology presence or absence [32, 33].

Ultrasound and OI are both non-invasive technologies. Thus, researchers can measure various aspects of the tumor in a dynamic way, improving the quality of experimental data and reducing the number of mice needed to produce statistically valid results. These two tools have different characteristics: experimental ultrasound is a smaller scale version of a well-established clinical imaging technique, while experimental OI is only suitable for imaging preclinical models. Both experimental ultrasound and OI are operator-dependent; however, in our experiments, ultrasound and OI findings were highly concordant. Furthermore, histopathology validated the diagnostic role of these experimental imaging procedures, confirming positive and negative findings of both techniques.

Of note, the settings of the syngeneic murine model and immunogenic surrender with their implications, suggesting a novel mechanism that could explain MM rejection in BoxA-treated mice, have been recently reported [13, 33].

In conclusion, in our preclinical experience in a murine model, BoxA, the antagonist of HMGB1, exerted a protective role on MM. BoxA may represent a newfound immunotherapeutic strategy against cancer, although further preclinical studies are needed to confirm these preliminary findings before translating BoxA to a clinical model. The combined use of ultrasound and OI allowed the detection of the onset and growth of tumors, providing a resourceful tool to follow the development of the disease in each animal, maintaining experimental homogeneity, fulfilling the requirement to eliminate “animal discomfort” by detecting critical situations leading to compassionate euthanasia, and potentially decreasing the number of animals needed.

## Data Availability

Data and materials can be provided on request.

## Abbreviations

HBMG1: High mobility group box 1
MM: Malignant mesothelioma
MRI: Magnetic resonance imaging
OI: Optical imaging
